# Supplementary material for: Randomized double-blind placebo-controlled proof-of-concept trial of resveratrol for outpatient treatment of mild coronavirus disease (COVID-19)
Source: Sci Rep. 2022 Jun 29;12:10978. doi: 10.1038/s41598-022-13920-9 (PMC9243086; doi:10.1038/s41598-022-13920-9)
Supplement: Supplementary file 1 — Supplementary Information. [file 41598_2022_13920_MOESM1_ESM.docx]

Supplemental Tables for

**Randomized double-blind placebo-controlled proof-of-concept trial of resveratrol
for outpatient treatment of mild coronavirus disease (COVID-19)**

Marvin R. McCreary, Patrick M. Schnell, and Dale A. Rhoda

Correspondence: [Marvin.McCreary+RCT@gmail.com](mailto:Marvin.McCreary+RCT@gmail.com)

**Supplementary Materials:** The tables listed below are available on the following pages.

- Table S-1. Symptoms at enrollment, by study group
- Table S-2a. Symptoms from daily diary - day 1, by study group
- Table S-2b. Symptoms from daily diary - days 2-15 & 21, by study group
- Table S-3a. Symptoms over the past week, asked on day 1, by study group
- Table S-3b. Symptoms over the past week, asked on days 8, 15 and 21, by study group
- Table S-4. Analysis of risk ratios among subgroups of study participants

| Table S-1. Symptoms at enrollment, by study group | | |  |  |  |
| --- | --- | --- | --- | --- | --- |
|  |  |  |  |  |  |
| Symptom | Placebo  Group N | Resveratrol  Group N | Placebo Group  Had Symptom:  N (%) | Resveratrol Group Had Symptom:  N (%) | p-value |
| Fever > 100.4F (38C) | 50 | 48 | 17 (34.0) | 16 (33.3) | 1.000 |
| Felt feverish | 48 | 46 | 31 (64.6) | 28 (60.9) | 0.831 |
| Chills | 50 | 49 | 25 (50.0) | 24 (49.0) | 1.000 |
| Muscle aches | 51 | 47 | 38 (74.5) | 32 (68.1) | 0.510 |
| Runny nose | 47 | 43 | 28 (59.6) | 26 (60.5) | 1.000 |
| Sore throat | 47 | 44 | 25 (53.2) | 19 (43.2) | 0.403 |
| Cough | 48 | 47 | 41 (85.4) | 35 (74.5) | 0.208 |
| Shortness of breath | 46 | 43 | 8 (17.4) | 10 (23.3) | 0.600 |
| Nausea or vomiting | 47 | 43 | 8 (17.0) | 10 (23.3) | 0.599 |
| Headache | 50 | 47 | 35 (70.0) | 34 (72.3) | 0.826 |
| Abdominal pain | 47 | 40 | 7 (14.9) | 4 (10.0) | 0.537 |
| Diarrhea | 47 | 44 | 16 (34.0) | 14 (31.8) | 1.000 |
| p-values from Fisher's exact test, not adjusted for multiple comparisons | | | | | |

| Table S-2a. Symptoms from daily diary - day 1, by study group | | |  |  |  |  |  |  |
| --- | --- | --- | --- | --- | --- | --- | --- | --- |
|  |  |  |  |  |  |  |  |  |
|  | Placebo  N | Resveratrol  N | Reported 1+ on Day 1 | | | Reported 3+ on Day 1 | | |
|  |  |  | Placebo  N (%) | Resveratrol N (%) | p-value | Placebo  N (%) | Resveratrol N (%) | p-value |
| Shortness of breath - Severity | 49 | 50 | 17 (34.7) | 18 (36.0) | 1.000 | 0 (0.0) | 1 (2.0) | 1.000 |
| Shortness of breath - ADL Interference | 49 | 50 | 49 (100.0) | 50 (100.0) | 1.000 | 5 (10.2) | 5 (10.0) | 1.000 |
| Fatigue - Severity | 49 | 50 | 45 (91.8) | 45 (90.0) | 1.000 | 8 (16.3) | 8 (16.0) | 1.000 |
| Fatigue - ADL Interference | 49 | 50 | 49 (100.0) | 50 (100.0) | 1.000 | 28 (57.1) | 24 (48.0) | 0.423 |
| Diarrhea - Frequency | 15 | 11 | 14 (93.3) | 11 (100.0) | 1.000 | 4 (26.7) | 4 (36.4) | 0.683 |
| ADL = activities of daily living |  |  |  |  |  |  |  |  |
| p-value from Fisher's exact test, not adjusted for multiple comparisons | | | | | | | | |
|  |  |  |  |  |  |  |  |  |
|  |  |  |  |  |  |  |  |  |
| Table S-2b. Symptoms from daily diary - days 2-15 & 21, by study group | | | |  |  |  |  |  |
|  |  |  |  |  |  |  |  |  |
|  | Placebo  N | Resveratrol  N | Ever reported 1+ after Day 1 | | | Ever reported 3+ after Day 1 | | |
|  |  |  | Placebo  N (%) | Resveratrol N (%) | p-value | Placebo  N (%) | Resveratrol N (%) | p-value |
| Shortness of breath - Severity | 50 | 49 | 28 (56.0) | 24 (49.0) | 0.548 | 2 (4.0) | 2 (4.1) | 1.000 |
| Shortness of breath - ADL Interference | 50 | 49 | 50 (100.0) | 49 (100.0) | 1.000 | 12 (24.0) | 7 (14.3) | 0.308 |
| Fatigue - Severity | 50 | 49 | 47 (94.0) | 48 (98.0) | 0.617 | 9 (18.0) | 14 (28.6) | 0.241 |
| Fatigue - ADL Interference | 50 | 49 | 50 (100.0) | 49 (100.0) | 1.000 | 31 (62.0) | 26 (53.1) | 0.419 |
| Diarrhea - Frequency | 17 | 40 | 17 (100.0) | 40 (100.0) | 1.000 | 4 (23.5) | 14 (35.0) | 0.537 |
| ADL = activities of daily living |  |  |  |  |  |  |  |  |
| p-values from Fisher's exact test, not adjusted for multiple comparisons | | | |  |  |  |  |  |

| Table S-3a. Symptoms over the past week, asked on day 1, by study group | | | | | | |  |  |  |
| --- | --- | --- | --- | --- | --- | --- | --- | --- | --- |
|  |  |  |  |  |  |  |  |  |  |
|  |  | Placebo  N | Resveratrol N | Reported 1+ on Day 1 | | | Reported 3+ on Day 1 | | |
|  |  |  |  | Placebo  N (%) | Resveratrol N (%) | p-value | Placebo  N (%) | Resveratrol N (%) | p-value |
| Increased flatulence | Presence | 45 | 46 | 11 (24.4) | 15 (32.6) | 0.488 |  |  |  |
| Rash | Presence | 45 | 45 | 6 (13.3) | 0 (0.0) | **0.026** |  |  |  |
| Hives | Presence | 44 | 46 | 3 (6.8) | 0 (0.0) | 0.113 |  |  |  |
| Bruise easily | Presence | 45 | 46 | 3 (6.7) | 1 (2.2) | 0.361 |  |  |  |
| Dry mouth | Severity | 45 | 46 | 25 (55.6) | 30 (65.2) | 0.395 | 4 (8.9) | 1 (2.2) | 0.203 |
| Difficulty swallowing | Severity | 45 | 46 | 11 (24.4) | 10 (21.7) | 0.807 | 1 (2.2) | 2 (4.3) | 1.000 |
| Sore throat / mouth | Severity | 45 | 46 | 29 (64.4) | 26 (56.5) | 0.522 | 1 (2.2) | 3 (6.5) | 0.617 |
| Hoarse voice | Severity | 45 | 46 | 28 (62.2) | 26 (56.5) | 0.671 | 1 (2.2) | 1 (2.2) | 1.000 |
| Problems tasting/smelling | Severity | 45 | 46 | 20 (44.4) | 29 (63.0) | 0.094 | 9 (20.0) | 11 (23.9) | 0.801 |
| Decreased appetite | Severity | 45 | 46 | 29 (64.4) | 40 (87.0) | **0.015** | 4 (8.9) | 11 (23.9) | 0.088 |
| Nausea | Frequency | 45 | 46 | 19 (42.2) | 23 (50.0) | 0.530 | 4 (8.9) | 2 (4.3) | 0.434 |
|  | Severity | 19 | 23 | 18 (94.7) | 22 (95.7) | 1.000 | 3 (15.8) | 2 (8.7) | 0.644 |
| Vomiting | Frequency | 45 | 46 | 6 (13.3) | 2 (4.3) | 0.158 | 1 (2.2) | 0 (0.0) | 0.495 |
|  | Severity | 19 | 23 | 5 (26.3) | 2 (8.7) | 0.214 | 1 (5.3) | 0 (0.0) | 0.452 |
| Heartburn | Frequency | 45 | 46 | 19 (42.2) | 15 (32.6) | 0.391 | 3 (6.7) | 1 (2.2) | 0.361 |
|  | Severity | 19 | 15 | 19 (100.0) | 15 (100.0) | 1.000 | 2 (10.5) | 0 (0.0) | 0.492 |
| Bloating | Frequency | 45 | 46 | 16 (35.6) | 23 (50.0) | 0.205 | 2 (4.4) | 4 (8.7) | 0.677 |
|  | Severity | 16 | 23 | 14 (87.5) | 20 (87.0) | 1.000 | 0 (0.0) | 1 (4.3) | 1.000 |
| Diarrhea | Frequency | 22 | 28 | 10 (45.5) | 18 (64.3) | 0.253 | 2 (9.1) | 2 (7.1) | 1.000 |
| Constipation | Severity | 45 | 46 | 9 (20.0) | 6 (13.0) | 0.410 | 0 (0.0) | 1 (2.2) | 1.000 |
| Pain in abdomen | Frequency | 45 | 46 | 13 (28.9) | 17 (37.0) | 0.505 | 5 (11.1) | 0 (0.0) | **0.026** |
|  | Severity | 13 | 17 | 12 (92.3) | 17 (100.0) | 0.433 | 2 (15.4) | 0 (0.0) | 0.179 |
|  | ADL Interference | 13 | 17 | 13 (100.0) | 17 (100.0) | 1.000 | 6 (46.2) | 2 (11.8) | **0.049** |
| Cough | Severity | 45 | 46 | 41 (91.1) | 38 (82.6) | 0.354 | 8 (17.8) | 4 (8.7) | 0.231 |
|  | ADL Interference | 41 | 38 | 41 (100.0) | 38 (100.0) | 1.000 | 11 (26.8) | 10 (26.3) | 1.000 |
| Wheezing | Severity | 45 | 46 | 14 (31.1) | 13 (28.3) | 0.821 | 1 (2.2) | 0 (0.0) | 0.495 |
| Racing heartbeat | Frequency | 45 | 46 | 15 (33.3) | 15 (32.6) | 1.000 | 3 (6.7) | 1 (2.2) | 0.361 |
| Itchy skin | Severity | 45 | 46 | 6 (13.3) | 5 (10.9) | 0.758 | 0 (0.0) | 0 (0.0) | 1.000 |
| Dizziness | Severity | 45 | 46 | 18 (40.0) | 15 (32.6) | 0.517 | 1 (2.2) | 1 (2.2) | 1.000 |
| Blurry vision | Severity | 45 | 46 | 13 (28.9) | 10 (21.7) | 0.477 | 1 (2.2) | 0 (0.0) | 0.495 |
|  | ADL Interference | 13 | 10 | 13 (100.0) | 10 (100.0) | 1.000 | 3 (23.1) | 2 (20.0) | 1.000 |
| Redness or watery eyes | Severity | 45 | 46 | 17 (37.8) | 21 (45.7) | 0.526 | 2 (4.4) | 2 (4.3) | 1.000 |
| Problems concentrating | Severity | 45 | 46 | 22 (48.9) | 26 (56.5) | 0.531 | 1 (2.2) | 4 (8.7) | 0.361 |
|  | ADL Interference | 22 | 26 | 22 (100.0) | 26 (100.0) | 1.000 | 7 (31.8) | 10 (38.5) | 0.765 |
| Problems with memory | Severity | 45 | 46 | 9 (20.0) | 14 (30.4) | 0.336 | 0 (0.0) | 2 (4.3) | 0.495 |
|  | ADL Interference | 9 | 14 | 9 (100.0) | 14 (100.0) | 1.000 | 1 (11.1) | 3 (21.4) | 1.000 |
| Pain | Frequency | 44 | 46 | 27 (61.4) | 34 (73.9) | 0.261 | 11 (25.0) | 12 (26.1) | 1.000 |
|  | Severity | 27 | 34 | 26 (96.3) | 34 (100.0) | 0.443 | 5 (18.5) | 6 (17.6) | 1.000 |
|  | ADL Interference | 27 | 34 | 27 (100.0) | 34 (100.0) | 1.000 | 13 (48.1) | 13 (38.2) | 0.603 |
| Headache | Frequency | 45 | 46 | 40 (88.9) | 41 (89.1) | 1.000 | 18 (40.0) | 13 (28.3) | 0.274 |
|  | Severity | 40 | 41 | 39 (97.5) | 39 (95.1) | 1.000 | 10 (25.0) | 8 (19.5) | 0.601 |
|  | ADL Interference | 40 | 41 | 40 (100.0) | 41 (100.0) | 1.000 | 16 (40.0) | 17 (41.5) | 1.000 |
|  |  | Placebo  N | Resveratrol N | Reported 1+ on Day 1 | | | Reported 3+ on Day 1 | | |
|  |  |  |  | Placebo  N (%) | Resveratrol N (%) | p-value | Placebo  N (%) | Resveratrol N (%) | p-value |
| Aching muscles | Frequency | 45 | 46 | 39 (86.7) | 39 (84.8) | 1.000 | 18 (40.0) | 16 (34.8) | 0.668 |
|  | Severity | 39 | 39 | 39 (100.0) | 38 (97.4) | 1.000 | 6 (15.4) | 10 (25.6) | 0.401 |
|  | ADL Interference | 37 | 39 | 37 (100.0) | 39 (100.0) | 1.000 | 18 (48.6) | 21 (53.8) | 0.819 |
| Aching joints | Frequency | 45 | 46 | 34 (75.6) | 29 (63.0) | 0.257 | 6 (13.3) | 9 (19.6) | 0.574 |
|  | Severity | 34 | 29 | 32 (94.1) | 27 (93.1) | 1.000 | 4 (11.8) | 6 (20.7) | 0.492 |
|  | ADL Interference | 33 | 29 | 33 (100.0) | 29 (100.0) | 1.000 | 7 (21.2) | 10 (34.5) | 0.269 |
| Nosebleeds | Frequency | 45 | 46 | 2 (4.4) | 5 (10.9) | 0.434 | 0 (0.0) | 0 (0.0) | 1.000 |
|  | Severity | 2 | 5 | 2 (100.0) | 5 (100.0) | 1.000 | 0 (0.0) | 0 (0.0) | 1.000 |
| Shivering | Frequency | 45 | 46 | 28 (62.2) | 24 (52.2) | 0.399 | 7 (15.6) | 7 (15.2) | 1.000 |
|  | Severity | 28 | 24 | 27 (96.4) | 24 (100.0) | 1.000 | 4 (14.3) | 5 (20.8) | 0.716 |
| ADL = activities of daily living | |  |  |  |  |  |  |  |  |
| p-value from Fisher's exact test, not adjusted for multiple comparisons | | | | | | | |  |  |
| Shaded cells are p-values below 0.05 | |  |  |  |  |  |  |  |  |

| Table S-3b. Symptoms over the past week, asked on days 8, 15 and 21, by study group | | | | | | | | | | |
| --- | --- | --- | --- | --- | --- | --- | --- | --- | --- | --- |
|  | |  |  |  |  |  |  |  |  |  |
|  | |  | Placebo  N | Resveratrol N | Ever reported 1+ after Day 1 | | | Ever reported 3+ after Day 1 | | |
|  | |  |  |  | Placebo  N (%) | Resveratrol N (%) | p-value | Placebo  N (%) | Resveratrol N (%) | p-value |
| Increased flatulence | | Presence | 35 | 39 | 16 (45.7) | 20 (51.3) | 0.650 |  |  |  |
| Rash | | Presence | 35 | 39 | 5 (14.3) | 6 (15.4) | 1.000 |  |  |  |
| Hives | | Presence | 35 | 39 | 2 (5.7) | 5 (12.8) | 0.435 |  |  |  |
| Bruise easily | | Presence | 35 | 39 | 5 (14.3) | 3 (7.7) | 0.464 |  |  |  |
| Dry mouth | | Severity | 35 | 39 | 21 (60.0) | 28 (71.8) | 0.331 | 4 (11.4) | 0 (0.0) | **0.046** |
| Difficulty swallowing | | Severity | 35 | 39 | 9 (25.7) | 6 (15.4) | 0.386 | 0 (0.0) | 0 (0.0) | 1.000 |
| Sore throat / mouth | | Severity | 35 | 39 | 17 (48.6) | 18 (46.2) | 1.000 | 0 (0.0) | 1 (2.6) | 1.000 |
| Hoarse voice | | Severity | 35 | 39 | 20 (57.1) | 19 (48.7) | 0.494 | 1 (2.9) | 0 (0.0) | 0.473 |
| Problems tasting/smelling | | Severity | 35 | 39 | 24 (68.6) | 30 (76.9) | 0.445 | 9 (25.7) | 15 (38.5) | 0.321 |
| Decreased appetite | | Severity | 35 | 39 | 26 (74.3) | 29 (74.4) | 1.000 | 3 (8.6) | 3 (7.7) | 1.000 |
| Nausea | | Frequency | 35 | 39 | 17 (48.6) | 24 (61.5) | 0.350 | 2 (5.7) | 9 (23.1) | **0.050** |
|  |  | Severity | 17 | 24 | 17 (100.0) | 24 (100.0) | 1.000 | 0 (0.0) | 0 (0.0) | 1.000 |
| Vomiting | | Frequency | 35 | 39 | 3 (8.6) | 2 (5.1) | 0.662 | 0 (0.0) | 0 (0.0) | 1.000 |
|  |  | Severity | 17 | 24 | 3 (17.6) | 2 (8.3) | 0.633 | 0 (0.0) | 0 (0.0) | 1.000 |
| Heartburn | | Frequency | 35 | 39 | 14 (40.0) | 19 (48.7) | 0.490 | 4 (11.4) | 4 (10.3) | 1.000 |
|  |  | Severity | 14 | 19 | 14 (100.0) | 19 (100.0) | 1.000 | 0 (0.0) | 1 (5.3) | 1.000 |
| Bloating | | Frequency | 35 | 39 | 19 (54.3) | 27 (69.2) | 0.233 | 2 (5.7) | 4 (10.3) | 0.677 |
|  |  | Severity | 19 | 27 | 18 (94.7) | 27 (100.0) | 0.413 | 1 (5.3) | 2 (7.4) | 1.000 |
| Diarrhea | | Frequency | 31 | 31 | 19 (61.3) | 27 (87.1) | **0.040** | 4 (12.9) | 9 (29.0) | 0.211 |
| Constipation | | Severity | 35 | 39 | 14 (40.0) | 14 (35.9) | 0.812 | 1 (2.9) | 1 (2.6) | 1.000 |
| Pain in abdomen | | Frequency | 35 | 39 | 12 (34.3) | 19 (48.7) | 0.244 | 1 (2.9) | 3 (7.7) | 0.617 |
|  |  | Severity | 12 | 19 | 12 (100.0) | 19 (100.0) | 1.000 | 0 (0.0) | 2 (10.5) | 0.510 |
|  |  | ADL Interference | 12 | 19 | 12 (100.0) | 19 (100.0) | 1.000 | 4 (33.3) | 5 (26.3) | 0.704 |
| Cough | | Severity | 35 | 39 | 31 (88.6) | 30 (76.9) | 0.231 | 7 (20.0) | 3 (7.7) | 0.176 |
|  |  | ADL Interference | 31 | 30 | 31 (100.0) | 30 (100.0) | 1.000 | 9 (29.0) | 8 (26.7) | 1.000 |
| Wheezing | | Severity | 34 | 39 | 8 (23.5) | 13 (33.3) | 0.441 | 3 (8.8) | 1 (2.6) | 0.333 |
| Racing heartbeat | | Frequency | 35 | 39 | 19 (54.3) | 16 (41.0) | 0.351 | 2 (5.7) | 2 (5.1) | 1.000 |
| Itchy skin | | Severity | 35 | 39 | 10 (28.6) | 9 (23.1) | 0.606 | 0 (0.0) | 1 (2.6) | 1.000 |
| Dizziness | | Severity | 35 | 39 | 16 (45.7) | 22 (56.4) | 0.485 | 2 (5.7) | 2 (5.1) | 1.000 |
| Blurry vision | | Severity | 35 | 39 | 10 (28.6) | 11 (28.2) | 1.000 | 1 (2.9) | 1 (2.6) | 1.000 |
|  |  | ADL Interference | 10 | 11 | 10 (100.0) | 11 (100.0) | 1.000 | 4 (40.0) | 4 (36.4) | 1.000 |
| Redness or watery eyes | | Severity | 35 | 39 | 16 (45.7) | 16 (41.0) | 0.815 | 0 (0.0) | 0 (0.0) | 1.000 |
| Problems concentrating | | Severity | 35 | 39 | 21 (60.0) | 19 (48.7) | 0.359 | 1 (2.9) | 1 (2.6) | 1.000 |
|  |  | ADL Interference | 21 | 19 | 21 (100.0) | 19 (100.0) | 1.000 | 5 (23.8) | 4 (21.1) | 1.000 |
| Problems with memory | | Severity | 35 | 39 | 12 (34.3) | 14 (35.9) | 1.000 | 1 (2.9) | 1 (2.6) | 1.000 |
|  |  | ADL Interference | 12 | 14 | 12 (100.0) | 14 (100.0) | 1.000 | 1 (8.3) | 3 (21.4) | 0.598 |
| Pain | | Frequency | 35 | 39 | 22 (62.9) | 26 (66.7) | 0.809 | 8 (22.9) | 2 (5.1) | **0.040** |
|  |  | Severity | 22 | 26 | 22 (100.0) | 26 (100.0) | 1.000 | 2 (9.1) | 3 (11.5) | 1.000 |
|  |  | ADL Interference | 22 | 26 | 22 (100.0) | 26 (100.0) | 1.000 | 6 (27.3) | 6 (23.1) | 0.751 |
| Headache | | Frequency | 35 | 39 | 30 (85.7) | 31 (79.5) | 0.552 | 13 (37.1) | 12 (30.8) | 0.627 |
|  |  | Severity | 30 | 31 | 30 (100.0) | 31 (100.0) | 1.000 | 3 (10.0) | 5 (16.1) | 0.707 |
|  |  | ADL Interference | 30 | 31 | 30 (100.0) | 31 (100.0) | 1.000 | 8 (26.7) | 11 (35.5) | 0.582 |
|  |  | | Placebo  N | Resveratrol N | Reported 1+ after Day 1 | | | Reported 3+ after Day 1 | | |
|  |  | |  |  | Placebo  N (%) | Resveratrol N (%) | p-value | Placebo  N (%) | Resveratrol N (%) | p-value |
| Aching muscles | | Frequency | 35 | 39 | 24 (68.6) | 26 (66.7) | 1.000 | 11 (31.4) | 6 (15.4) | 0.165 |
|  |  | Severity | 24 | 26 | 24 (100.0) | 26 (100.0) | 1.000 | 2 (8.3) | 3 (11.5) | 1.000 |
|  |  | ADL Interference | 24 | 26 | 24 (100.0) | 26 (100.0) | 1.000 | 8 (33.3) | 5 (19.2) | 0.339 |
| Aching joints | | Frequency | 35 | 39 | 20 (57.1) | 20 (51.3) | 0.647 | 2 (5.7) | 3 (7.7) | 1.000 |
|  |  | Severity | 20 | 20 | 19 (95.0) | 19 (95.0) | 1.000 | 1 (5.0) | 2 (10.0) | 1.000 |
|  |  | ADL Interference | 20 | 20 | 20 (100.0) | 20 (100.0) | 1.000 | 4 (20.0) | 3 (15.0) | 1.000 |
| Nosebleeds | | Frequency | 35 | 39 | 2 (5.7) | 5 (12.8) | 0.435 | 0 (0.0) | 0 (0.0) | 1.000 |
|  |  | Severity | 2 | 5 | 2 (100.0) | 5 (100.0) | 1.000 | 0 (0.0) | 0 (0.0) | 1.000 |
| Shivering | | Frequency | 35 | 39 | 14 (40.0) | 12 (30.8) | 0.469 | 2 (5.7) | 2 (5.1) | 1.000 |
|  |  | Severity | 14 | 12 | 14 (100.0) | 12 (100.0) | 1.000 | 2 (14.3) | 2 (16.7) | 1.000 |
| ADL = activities of daily living | | |  |  |  |  |  |  |  |  |
| p-value from Fisher's exact test, not adjusted for multiple comparisons | | | | | | | |  |  |  |
| Shaded cells are p-values at or below 0.05 | | | |  |  |  |  |  |  |  |

| Table S-4. Analysis of risk ratios among subgroups of study participants | | | | |  |  |  |
| --- | --- | --- | --- | --- | --- | --- | --- |
|  |  |  |  |  |  |  |  |
| Outcome | Subgroup | N with outcome in resveratrol group | N in resveratrol group where outcome is not missing | N with outcome in placebo group | N in placebo group where outcome is not missing | Risk ratio | p-value |
| Hospitalization | All | 1 | 50 | 3 | 50 | 0.33 | 0.617 |
|  | Male | 1 | 21 | 1 | 18 | 0.86 | 1.000 |
|  | Female | 0 | 29 | 2 | 32 | 0.00 | 0.493 |
|  | Race: White | 1 | 45 | 2 | 44 | 0.49 | 0.616 |
|  | On ACE controller | 1 | 5 | 2 | 9 | 0.90 | 1.000 |
|  | Not on ACE controller | 0 | 45 | 1 | 41 | 0.00 | 0.477 |
|  | Age < 65 | 1 | 41 | 2 | 43 | 0.52 | 1.000 |
|  | Age 65+ | 0 | 9 | 1 | 7 | 0.00 | 0.438 |
| Pneumonia | All | 4 | 50 | 8 | 50 | 0.50 | 0.357 |
|  | Male | 3 | 21 | 1 | 18 | 2.57 | 0.609 |
|  | Female | 1 | 29 | 7 | 32 | 0.16 | 0.055 |
|  | Race: White | 3 | 45 | 7 | 44 | 0.42 | 0.197 |
|  | On ACE controller | 1 | 5 | 3 | 9 | 0.60 | 1.000 |
|  | Not on ACE controller | 3 | 45 | 5 | 41 | 0.55 | 0.470 |
|  | Age < 65 | 4 | 41 | 5 | 43 | 0.84 | 1.000 |
|  | Age 65+ | 0 | 9 | 3 | 7 | 0.00 | 0.063 |
| Pulmonary embolism | All | 1 | 50 | 1 | 49 | 0.98 | 1.000 |
|  | Male | 1 | 21 | 0 | 18 | NA | NA |
|  | Female | 0 | 29 | 1 | 31 | 0.00 | 1.000 |
|  | Race: White | 0 | 45 | 1 | 43 | 0.00 | 0.489 |
|  | On ACE controller | 0 | 5 | 0 | 9 | NA | NA |
|  | Not on ACE controller | 1 | 45 | 1 | 40 | 0.89 | 1.000 |
|  | Age < 65 | 1 | 41 | 0 | 42 | NA | NA |
|  | Age 65+ | 0 | 9 | 1 | 7 | 0.00 | 0.438 |
| Emergency Room Visit for COVID | All | 4 | 50 | 7 | 50 | 0.57 | 0.525 |
|  | Male | 3 | 21 | 1 | 18 | 2.57 | 0.609 |
|  | Female | 1 | 29 | 6 | 32 | 0.18 | 0.106 |
|  | Race: White | 3 | 45 | 6 | 44 | 0.49 | 0.315 |
|  | On ACE controller | 1 | 5 | 3 | 9 | 0.60 | 1.000 |
|  | Not on ACE controller | 3 | 45 | 4 | 41 | 0.68 | 0.704 |
|  | Age < 65 | 4 | 41 | 4 | 43 | 1.05 | 1.000 |
|  | Age 65+ | 0 | 9 | 3 | 7 | 0.00 | 0.063 |
| NA = not applicable; ACE = Angiotensin-converting enzyme  p-values not adjusted for multiple comparisons | | | |  |  |  |  |
